# Growth of freshwater cyanobacterium *Aphanizomenon* sp. ULC602 in different growing and nutrient conditions

**DOI:** 10.3389/fmicb.2023.1220818

**Published:** 2023-12-21

**Authors:** Mai-Lan Pham, Elahe Askarzadmohassel, Martin Brandl

**Affiliations:** Center for Water and Environmental Sensors, Department for Integrated Sensor Systems, University for Continuing Education Krems, Krems an der Donau, Austria

**Keywords:** cyanobacteria, *Aphanizomenon*, chlorophyll-a, freshwater, growing conditions

## Abstract

*Aphanizomenon* sp. ULC602, recently isolated in a Belgian lake, is a filamentous, nitrogen-fixing, freshwater cyanobacterium that is one of the primary producers of cyanotoxins following its bloom formation, causing water contamination. This study aims to evaluate the effects of growing conditions and essential nutrients on the growth of *Aphanizomenon* sp. ULC602 via its production of chlorophyll-a (Chlo-a). Our results indicated that this bacterium could grow well at temperatures ranging from 18 to 25°C with an optimal pH of 6.0–7.5 under continuous lighting. It grew slowly in the absence of a carbon source or at lower carbon concentrations. The addition of nitrogen from nitrate and urea led to a less than 50% reduction of Chlo-a content compared to the medium lacking nitrogen. The iron bioavailability significantly stimulated the Chlo-a production, but it was saturated by an iron concentration of 0.115 mM. Moreover, a decrease in Chlo-a biomass was observed under sulfur deficiency. The bacterium could not grow well in media containing various phosphorus sources. In conclusion, as the growth and consequent forming bloom of cyanobacteria can be stimulated or inhibited by environmental conditions and eutrophication, our investigation could contribute to further studies to control the blooming of the target bacterium in freshwater.

## 1 Introduction

Cyanobacteria, known as blue-green algae, are gram-negative photosynthetic bacteria that can inhabit both freshwater and marine ecosystems. They have the ability to flourish in extreme environmental conditions and adapt to various stress parameters including high light intensity, UV, extreme temperatures, high salt, and both low and high pH (Kultschar and Llewellyn, 2018). Generally, based on their morphology, this bacterial group can be unicellular, such as *Microcystis* sp. and *Synechococcus* sp., or filamentous, including *Anabaena* sp., *Aphanizomenon* sp., and *Oscillatoria* sp. (Chorus and Welker, 2021; Mehdizadeh Allaf and Peerhossaini, 2022). In addition, the blooming of cyanobacteria in waterbodies affects the water quality and can cause odor problems (Rastogi et al., 2015). Following the formation of bloom, most cyanobacteria also produce toxic compounds, cyanotoxins, as their secondary metabolites, such as microcystins, cylindrospermopsins, anatoxin-a, saxitoxins, nodularins, and lyngbyatoxins, among others (Walve and Larsson, 2007; Moore et al., 2008; Rastogi et al., 2015; Kultschar and Llewellyn, 2018; Jeong et al., 2020). These toxins are usually released into the aquatic environment during cell death and lysis, leading to the death of aquatic organisms and causing effects on human health (Hallegraeff, 1993; Rastogi et al., 2015; Huisman et al., 2018). Therefore, it is necessary to characterize factors that may result in the immense expansion of these bacteria.

*Aphanizomenon* is a planktic genus of cyanobacteria that is found in freshwater and is one of the primary toxic bloom-forming cyanobacteria (De Figueiredo et al., 2011; Cirés and Ballot, 2016; Van Hassel et al., 2022). Belonging to the order Nostocales, in the absence of nitrogen, bacteria of this genus are able to differentiate into nitrogen-fixing cells, so-called heterocysts (Kumar et al., 2009; Singh and Montgomery, 2011; Cirés et al., 2013; Zhang et al., 2017). These cells are generally pale yellow with various shapes (spherical to cylindrical) and usually found in the intercalary or terminal position of the filaments. They contribute the fixed nitrogen to vegetative cells in the filament (Cirés and Ballot, 2016; Singh et al., 2020). They usually accumulate cyanophycin granules at the poles adjacent to vegetative cells where nitrogen is stored (Singh et al., 2020). Furthermore, they can differentiate into other specialized spore-like cells, akinetes, which allow these bacteria to survive in harsh conditions (Kaplan-Levy et al., 2010). The akinetes look bigger, possess a thicker cell wall than vegetative and heterocyst cells, and accumulate a large amount of food reserves and DNA (Kaplan-Levy et al., 2010; Cirés et al., 2013; Cirés and Ballot, 2016; Singh et al., 2020).

Many studies indicated that the development, morphotype, phenotype, metabolism, and differentiation of cyanobacteria, in general, depend on growing conditions including light quality, temperature, pH, UV exposure, and nutrient demands in the medium (Kaplan-Levy et al., 2010; Larson et al., 2018; Singh et al., 2020). For example, in *Anabaena* sp. UTEX 2576, nitrogen sources and iron levels in the growth medium were demonstrated as essential factors affecting the production of natural pigments and the consumption of other mineral elements, such as P, Ca, Mg, B, Mo, Zn, and Cu (Norena-Caro et al., 2021). Phosphate limitation in the growing medium could trigger the formation of akinetes in *Anabaena circinalis* (Kaplan-Levy et al., 2010). Larson et al. (2018) observed that the formation of a harmful bloom of several nitrogen-fixing cyanobacteria was influenced by phosphorus-iron colimitation. A bulky abundance of heterocysts of nitrogen-fixing cyanobacteria was observed in the media containing low nitrogen with high phosphorus and iron (Larson et al., 2018). This research group also pointed out that iron plays an important role in the development of cyanobacterial biomass (Larson et al., 2018). Previous studies on the effects of the abovementioned factors on members of the *Aphanizomenon* genus focused on four traditional morphological groups, namely, *Aph. flos-aquae, Aph. gracile. Aph. issatschenkoi*, and *Aph. volzii* (Rekar and Hindák, 2002; Komárek and Komárková, 2006; Preußel et al., 2009; Ballot et al., 2010; Üveges et al., 2012; Cirés and Ballot, 2016; Cirés et al., 2017; Chen et al., 2020; Wen et al., 2022). Cirés et al. (2013) indicated that the differentiation of akinetes of *Aphanizomenon ovalisporum* was dependent on environmental temperature, and the highest number of akinetes was found at 20°C. These authors also investigated the influence of temperature on the production of saxitoxin, a potent neurotoxin produced by *Aphanizomenon gracile* (Cirés et al., 2017). In another study, the responses of *Aphanizomenon* strains to levels of phosphorus and nitrogen were evaluated (De Figueiredo et al., 2011). The study revealed that concentrations of orthophosphate ≤0.3 mg/L inhibited the growth of all strains. Nitrate variation did not affect the *Aphanizomenon gracile* species, while the other species were significantly sensitive to the depletion of this source (De Figueiredo et al., 2011). Furthermore, the co-cultivation of *Aphanizomenon flos-aquae* and *Microcystis aeruginosa* highlighted that temperature played a key role in the succession of bloom between the two strains (Wen et al., 2022).

Chlorophyll-a (Chlo-a) is known as the photosynthetic pigment of phytoplankton (Huang et al., 2022). It has been considered one of the well-known water quality indicators and a biomass indicator of cyanobacteria (Borowitzka and Moheimani, 2013; Viso-Vázquez et al., 2021; Mudaliar and Pandya, 2023). This indicator was used for predictions of cyanobacterial blooms on the surface water of the A Baxe reservoir (Viso-Vázquez et al., 2021). Similarly, surface water quality in two wetlands of India was also assessed based on the Chlo-a concentration produced by cyanobacteria existing in the water, along with the dissolved oxygen content using multi-temporal Sentinel-2 images (Mudaliar and Pandya, 2023).

The present research work illustrated the influences of growing conditions and nutrients in laboratory conditions on the growth of a freshwater filamentous cyanobacterium, *Aphanizomenon* sp. ULC602, which was recently isolated from a Belgian lake. The goal of this study was achieved by investigating the production of Chlo-a by the target bacterium in different growing conditions, including temperatures, pH, and photoperiods. The impacts of major nutrient sources, including C, N, Fe, S, and P sources, on Chlo-a biomass were also evaluated using various forms and concentrations of these nutrients.

## 2 Materials and methods

### 2.1 Bacteria and chemicals

*Aphanizomenon* sp. ULC602 was obtained from the Belgian Coordinated Collection of Microorganisms (BCCM; Belgium) (Van Hassel et al., 2022) and was cultivated in a 50% dilution of BG110 medium, which was prepared following the manufacturer’s guidelines (Table 1) in laboratory room temperature (23 ± 1°C), which is denoted as RT in this study. The cultures were continuously illuminated with white light in a 24 h/0 h light/dark period. The culture was kept in the growing medium at RT for a period of 30 days. All chemicals used in this study were purchased from Carl Roth (Austria) with high quality unless stated otherwise.

**Table 1:** Component of 50% BG110 (Std) in 1 L (BCCM, Belgium).

Component	Final concentration (mM)
K_2_HPO_4_•3H_2_O	0.0876
MgSO_4_•7H_2_O	0.3115
CaCl_2_•2H_2_O	0.1224
EDTA-Mg-Na_2_	0.0013
Citric acid	0.0143
C_6_H_8_FeNO_7_	0.0113
Na_2_CO_3_	0.095
NaHCO_3_	0.8999
Microelements*	0.5 mL

*Trace metal mix A5 with Co (Sigma, St. Louis, USA).

### 2.2 Experiment design

The filamentous cyanobacterium *Aphanizomenon* sp. ULC602 was cultivated and maintained in 50 mL of fresh 50% BG110 medium, pH 7.0 at RT, and illuminated with white light in a 24 h/0 h light/dark period for 30 days. The growth of the bacterium was assessed by regularly measuring the chlorophyll-a (Chlo-a) concentrations using a Chlo-a assay (Zavřel et al., 2015).

#### 2.2.1 Effects of growing conditions

The influences of growing conditions were evaluated using three factors: pH, temperature, and photoperiod. For evaluation of the influence of pH values on the growth and Chlo-a production of *Aphanizomenon* sp. ULC602, the cell culture was grown at RT in a 24 h/0 h light/dark period in 50 mL of 50% BG110 media, adjusting the pH in the range of 4.0–9.0 using 0.1 N NaOH and 0.1 N HCl.

The effects of growing temperature were determined by cultivating the bacterium in 50 mL of 50% dilution of BG110 medium at different temperatures, including 4°C, 12°C, 18°C, RT, and 37°C, under exposure to continuous lighting (24 h/0 h light/dark).

For the photoperiod experiment, the bacterium was grown in a 50% dilution of BG110 medium at RT and illuminated using three different photoperiods: 24 h/0 h, 12 h/12 h, and 0 h/24 h light/dark.

#### 2.2.2 Effects of nutrients

For monitoring the effects of nutrients, the culture was cultivated at RT in various modified 50% BG110 media adjusted to pH 7.0 under continuous light (photoperiod 24 h/0 h light/dark); these media are named and listed in Table S1. The standard medium is 50% BG110 (Std) at pH 7.0, which is lacking a nitrogen source, and the iron source used in this medium was in the form of Fe^3+^ as ammonium ferric citrate (C_6_H_8_FeNO_7_).

The effects of iron were investigated by cultivating the bacterium in modified media M2 to M9 containing different concentrations of C_6_H_8_FeNO_7_ from 0 to 0.0227 mM, as well as other iron forms such as 0.000185 mM FeCl_3_ and 0.0359 mM FeSO_4_. Among them, the iron source was removed in the M2 medium; the original Fe (III) in the Std medium was replaced by other Fe (III) forms such as FeCl_3_ in M4; the Fe (III) was replaced by Fe (II) in M8, and the M9 medium contained the combination of both Fe (II) and Fe (III) forms. The impact of an organic chelating agent, EDTA-Na-Mg_2_, on bacterial growth was also studied by removing it in the M3 medium.

Media M10 and M11 were supplied with nitrogen from 8.8241 mM NaNO_3_ and 1.5 mM urea, respectively. The amount of inorganic carbon in the 50% BG110 medium, which is from Na_2_CO_3_ and NaHCO_3_, varied from 0 to 0.095 mM in media M12 and M13, respectively. The effects of limited sulfur in the media were investigated using media M14 to M17 with the concentration of MgSO_4_ ranging from 0 to 0.15 mM. The PO_4_^3−^ source was removed in media M18. Three formulations of PO_4_^3−^ were added to media M19 to M21, including 0.175 mM K_2_HPO_4_ combined with 0.65 mM KH_2_PO_4_ (M19), 0.018 mM NaH_2_PO_4_ (M20), and 1.45 mM K_2_HPO_4_ (M21).

All experiments mentioned above were conducted for 30 days, and samples were taken twice per week. The growth of *Aphanizomenon* sp. ULC602 was evaluated by measuring the variability of the concentration of produced Chlo-a during the cultivation. After 30 days, cells were collected for flow cytometry analysis.

### 2.3 Chlorophyll-a assay

The Chlo-a concentration was measured by Chlo-a assay using a spectrophotometer, following the protocol of Zavřel et al. (2015) with some modifications. Briefly, 1 mL of bacterial culture was harvested by centrifugation at 13,000 x g at room temperature for 5 min. Then, 1 mL of pre-cooled 100% methanol was added to the harvested cell pellets and mixed for 5 min by vortexing. The mixture was then incubated in the dark at 4°C for 20 min to extract the pigment. Subsequently, the mixture was centrifuged at 13,000 x g for 5 min at 4°C. The absorbance of the collected supernatant was measured at 665 nm using the Lambda Bio + Spectrophotometer (Perkin Elmer, USA). The 100% methanol was used as the blank, and its absorbance was spectrophotometrically measured at 720 nm. The concentration of Chlo-a was calculated according to the following equation (Zavřel et al., 2015):


(1) $$\mathrm{Chlo}-a\;\left(\mathrm{\mu g}/\mathrm{ml}\right)=12.9447\;x\;\left(\mathrm{Ab}s_{665}-\mathrm{Ab}s_{720}\right)$$

where Abs_665_ is the absorbance of the Chlo-a at 665 nm and Abs_720_ is the absorbance of the 100% methanol at 720 nm.

The relative Chlo-a biomass content (%) was calculated based on the Chlo-a produced by the bacteria grown in different growing and nutrient conditions compared to those in the standard medium (Std) and standard growing conditions. The Chlo-a produced by the bacterium grown in 50% BG110 (pH 7.0, photoperiod of 24 h/0 h light/dark, at RT) was expressed as 100%.

### 2.4 Flow cytometry analysis

For the flow cytometry analysis, 1 mL of the bacterium culture adjusted at an optical density of 750 nm (OD_750_) ~ 0.5 was collected by centrifugation at 6,000 x g at 4°C for 5 min and washed twice with phosphate-buffered saline (PBS) 1X, pH 7.4. The cells were then re-suspended in 200 μL of PBS 1X buffer, pH 7.4. The fluorescence of Chlo-a from the cell suspension was analyzed by flow cytometry using a CytoFLEX Flow Cytometer (Beckman Coulter, Bream, CA, USA) following the manufacturer’s instructions. The obtained data were analyzed and normalized using Flowjo v10.8.1 software (LLC, BD, USA).

### 2.5 Confocal microscopy observation

Microscopic preparation was conducted following the previous description with some modifications (Nairn, 1969; Pham et al., 2020). After 1 month of cultivation, 1 mL of cells, adjusted to OD_750_ ~ 0.5, was collected by centrifugation at 6,000 x g at 4°C for 5 min. Cells were washed twice with PBS 1X buffer, pH 7.4, then fixed with 4% paraformaldehyde dissolved in PBS 1X for 30 min at RT. Subsequently, the cells were washed with PBS 1X twice by centrifugation (at 6,000 x g, 4°C, 5 min) to completely remove the fixing solution. The pellets were then re-suspended in 100 μL of PBS 1X. For the preparation of microslides, 20 μL of fixed cells in PBS 1X were dropped on the high precious cover glass (Marienfield, USA) and left to air-dry. The dried trace was then stained with wheat germ agglutinin (WGA; Alexa Fluor 488 conjugated; Thermo Fisher Scientific) solution (diluted 1:200 in 1% PBS-BSA) for 15 min at RT following the manufacturer’s instructions. The trace was then washed four times with PBS 1X, pH 7.4. Then, 5 μL of a mounting solution containing 50% glycerol in PBS 1X was added to the trace on the cover glass (Pham et al., 2020). The cover glass was attached to the microscope slide and sealed by ROTI seal (Carl Roth). Cells were then observed under Leica DMi8 confocal microscopy (Leica) with excitation at 488 nm and emission at 496 nm for WGA staining, excitation at 543 nm and emission at 670–720 nm for chlorophyll-a, and emission at 650–660 nm for phycocyanin as described previously (Hernández Mariné et al., 2004; Grigoryeva and Chistyakova, 2020).

### 2.6 Statical analysis

All experiments and measurements were conducted in triplicate. Data analyses were carried out statistically by Sigma plot software (Systat Software Inc., USA), and one-way ANOVA was used to test for comparisons in the mean values of the treatment groups. The evaluation of significant differences between the mean of the standard medium and the means of the individual media was also performed by the Holm-Sidak post-test. The data are expressed as the mean ± standard deviation (SD) when appropriate.

## 3 Results

### 3.1 Growth measurement

**Figure 1:**
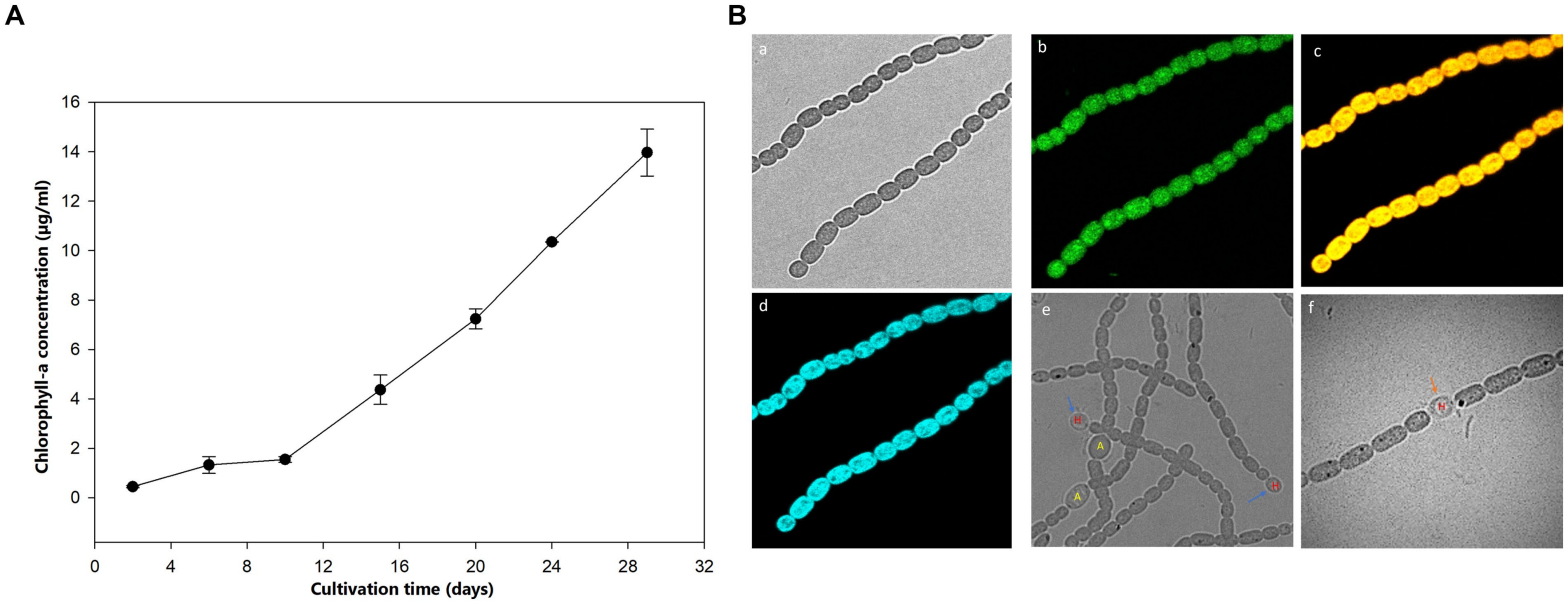
**(A)** Growth curve of *Aphanizomenon* sp. ULC602 after 30 days based on the production of Chlo-a. **(B)** Microscopic observation of *Aphanizomenon* sp. ULC602 by confocal laser scanning microscopy (CLSM): **(a)** transmission photomicrograph; **(b)** representing fluorescence from staining cells with wheat germ agglutinin (WGA) conjugated Alexa Fluor 488; **(c)** representing fluorescence from phycocyanin at 650–660 nm; **(d)** representing fluorescence from chlorophyll-a at 670–720 nm; **(e)** photomicrograph of heterocyst (H) and akinetes (A) cells; and **(f)** photomicrograph of heterocyst cell, which occurred in the intercalary position in the filament (H). Blue arrows show the heterocyst cells, while the black arrow indicates the akinetes cells.

The growth of *Aphanizomenon* sp. ULC602 was measured based on the production of Chlo-a by this bacterium, shown in Figure 1A. The cultivation was started with 1% pre-culture supplement in 50 mL of fresh 50% BG110 medium, pH 7.0, with a 24 h/0 h light/dark photoperiod at RT (Eq. 1). After the first 10 days, the Chlo-a biomass increased slightly, then rose remarkably over the following 20 days. After 1 month, the Chlo-a concentration reached 13.96 μg/mL. The morphology of this bacterium was imaged by confocal laser scanning microscopy using the natural pigment fluorescence (Chlo-a and phycocyanin) of the bacterium (Figures 1Bc). The extracellular polymeric substance (EPS) of the bacterium was observed by staining with WGA-Alexa Fluor 488 conjugated (Figures 1Ba). Results showed that a trichome of the studied strain, composed of plenty of single cells, was heterocystous and in straight or bent shapes (Figures 1Ba–f). A single vegetative cell was cylindrical to barrel-shaped. The heterocysts were spherical and found in the intercalary or terminal position in each trichome (Figures 1Be,f). The akinetes were oval and bigger than the vegetative and heterocyst cells (Figures 1Be).

### 3.2 Effects of growing conditions on the production of Chlo-a

**Figure 2:**
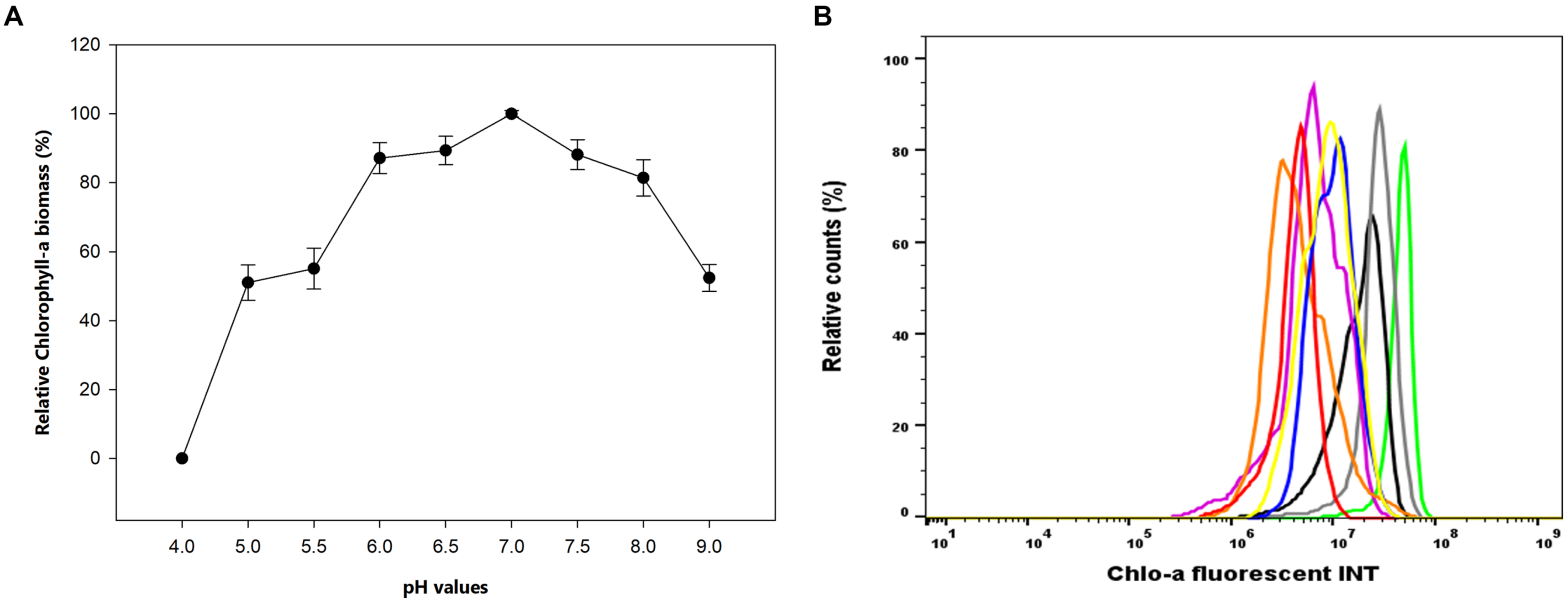
**(A)** Effects of pH on the production of Chlo-a of *Aphanizomenon* sp. ULC602. Data are presented as means ± SD (*n* = 3). Statistical analysis was performed using the one-way ANOVA method (*n* = 27, *F* = 160.711, *p* < 0.001). **(B)** Flow cytometry analysis of *Aphanizomenon* sp. ULC602 grown in media with various pH: pH 4.0: no data, pH 7.0 (green), pH 6.5 (grey), pH 6.0 (blue), pH 7.5 (black), pH 5.5 (pink), pH 5.0 (orange), pH 8.0 (yellow), and pH 9.0 (red).

The influence of pH values on the Chlo-a production in *Aphanizomenon* sp. ULC602 is illustrated in Figure 2A. The results showed that the bacterium can grow well at neutral pH ranging from 6.0 to 7.5, with the optimal pH value of 7.0. At the acidic pH range (from pH 4.0 to 5.5) and the basic pH of 9.0, the Chlo-a concentrations were significantly lower and accounted for approximately 50% of the Chlo-a concentration at pH 7.0. The bacterium was dead at pH 4.0. The Chlo-a fluorescence intensities in cells obtained from different pH samples were analyzed by flow cytometry with the Chlo-a intensity in the Std medium sample (green) as the reference (Figure 2B). Similarly, the intensities of Chlo-a gradually declined at acidic and basic pH.

The effects of growing temperatures and photoperiods are recorded in Table 2. The bacterium could grow well at 18°C for 1 month, similar to its growth at RT (Holm-Sidak test: *t* = 1.677, *p* = 0.132); however, at a lower temperature (12°C) and at a high temperature (37°C), the bacterium grew slowly, and the produced Chlo-a biomass only accounted for 43.97 and 25.65%, respectively, compared to the biomass obtained at RT (Holm-Sidak test: *t* = 19.659 and 23.489, respectively, *p* < 0.05). At the cold temperature (4°C), cells nearly could not grow. In addition, three conditions of photoperiods were applied to grow the bacterium in the Std medium at RT, namely, 24 h/0 h, 12 h/12 h, and 0 h/24 h (light/dark). Among them, the cells grew well under continuous lighting (24 h/0 h), and the produced Chlo-a biomass in this condition was considered 100%. When reducing the lighting time, the cells grew slowly (*n* = 9, *F* = 667.129, *p* < 0.001). The Chlo-a biomass obtained when the lighting time decreased to 12 h (12 h/12 h light/dark) was less than 75% compared to the amount obtained in continuous lighting conditions (Holm-Sidak test: *t* = 74.371, *p* < 0.05). In the dark photoperiod (0 h/24 h light/dark), the cells did not grow.

**Table 2:** Effects of temperature and photoperiod on the production of Chlo-a of *Aphanizomenon* sp. ULC602.

Relative chlorophyll-a biomass (%)							
Temperatures (°C)					Photoperiod (light/dark)		
4°C	12°C	18°C	RT (23°C)	37°C	24 h/0 h	12 h/12 h	0 h/24 h
5.16 ± 0.13	43.97 ± 3.81	93.65 ± 2.39	100	25.65 ± 0.458	100	30.529 ± 1.16	7.89 ± 0.076

The data are presented as means ± SD (*n* = 3) (*p* < 0.05).

### 3.3 Effects of nutrients

**Figure 3:**
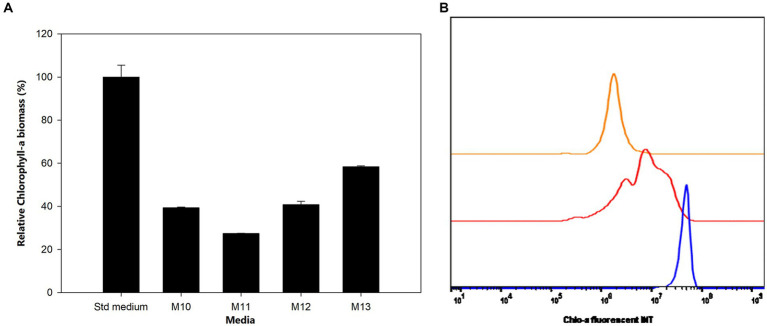
**(A)** Influence of carbon and nitrogen sources on the growth of *Aphanizomenon* sp. ULC602. Data are presented as means ± SD (*n* = 3). **(B)** Flow cytometry analysis of *Aphanizomenon* sp. ULC602 grown in media containing nitrogen sources: standard medium (Std; in blue), M10 containing 8.8241 mM NaNO_3_ (red), and M11 containing 1.5 mM urea (orange).

Figures 3A,B indicate the production of Chlo-a biomass by *Aphanizomenon* sp. ULC602 in modified media containing carbon and nitrogen sources. The Chlo-a biomass produced in the standard (Std) medium, 50% BG110, was expressed in relative biomass as 100%. In the presence of the nitrogen (N) sources of 8.8241 mM NaNO_3_ (M10) and 1.5 mM urea (M11), the Chlo-a biomass was reduced by less than 50% compared to the amount obtained in the Std medium (Holm-Sidak test: *t* = 28.654 and 34.285, respectively; *p* < 0.05). The Chlo-a-producing rate of the bacterium decreased following the reduction in the amount of inorganic carbon source in M12 and M13 (*t* = 27.965 and 19.657, *p* < 0.05).

**Figure 4:**
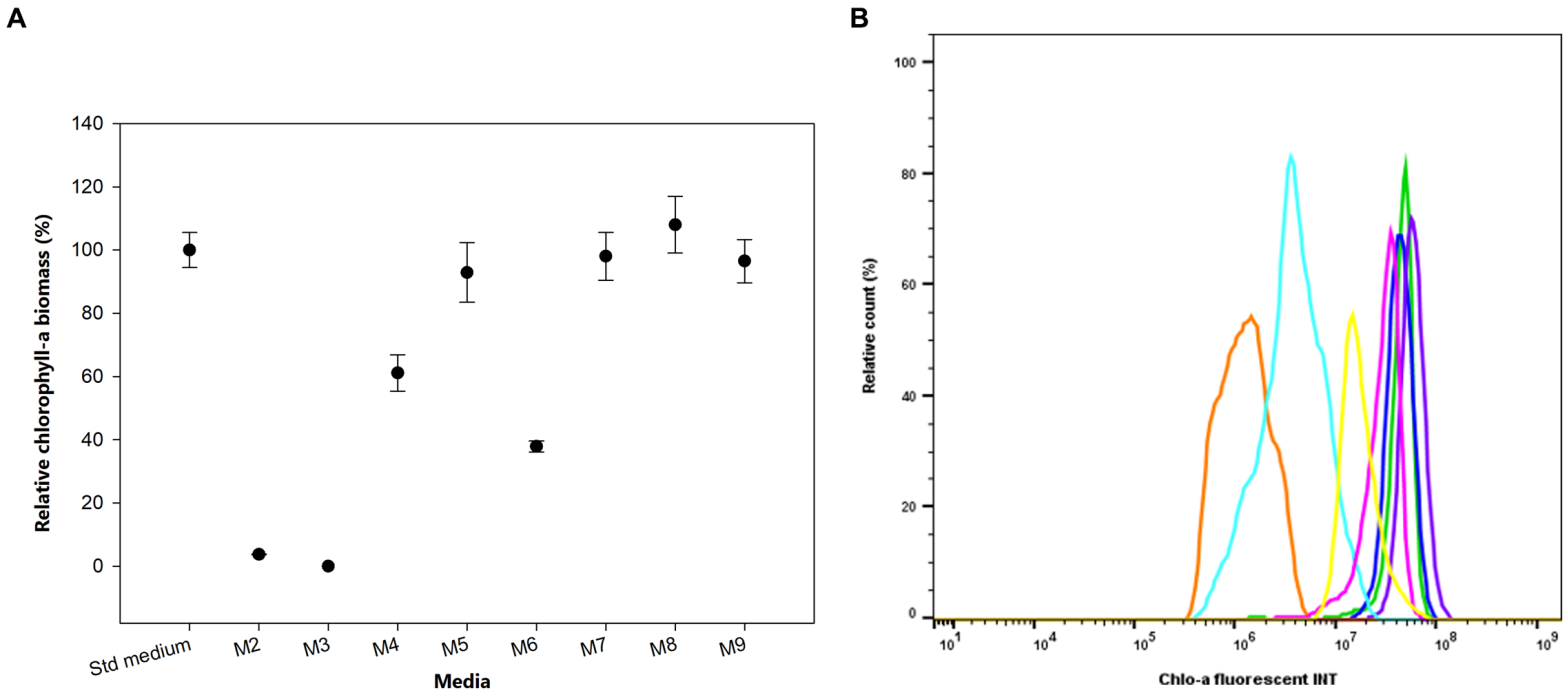
(**A**) Effects of iron sources on the production of Chlo-a of *Aphanizomenon* sp. ULC602. Data are presented as means ± SD (*n* = 3). Statistical analysis was performed using one-way ANOVA (*n* = 27, *F* = 145.182, *p* < 0.001). (**B**) Flow cytometry analysis of *Aphanizomenon* sp. ULC602 grown in iron-containing media. Std medium, 50% BG110 (green); M2, modified medium without iron (no data); M3, modified medium without iron and chelating agent EDTA (no data); M4, 0.0057 mM ammonium iron (III) citrate (light blue); M5, 0.0227 mM ammonium iron (III) citrate (yellow); M6, ammonium iron (III) citrate was replaced by 0.0002 mM FeCl_3_ (orange); M7, the medium with 0.0002 mM FeCl_3_ (blue); M8, ammonium iron (III) citrate was replaced by 0.0359 mM FeSO_4_ (purple); M9, the medium with 0.0359 mM FeSO_4_ (pink).

We investigated the growth and produced Chlo-a biomass of the bacterium in response to various iron (Fe) additions over 1 month, as exhibited in Figure 4A. The cells could not grow in the absence of soluble Fe^3+^ from ammonium iron citrate in the growing media (M2 and M3), especially when the chelating agent, EDTA, was simultaneously removed (M3) after 3 days of inoculation (Holman-Sidak test: *t* = 19.169 and 19.910, respectively; *p* < 0.05). The Chlo-a biomass declined to less than 60% in the M4 medium, which contained a half concentration of iron compared to the Std medium (Holman-Sidak test: *t* = 7.740; *p* < 0.05). The produced Chlo-a in the M7 medium, containing soluble Fe^3+^ from 0.0002 mM FeCl_3_ instead of ammonium iron citrate, was reduced to 36% of the concentration compared to the amount obtained in the Std medium (Holman-Sidak test: *t* = 12.368, *p* < 0.05). When increasing the iron concentration to 0.0227 mM in the M5 medium, cells produced a comparable amount of Chlo-a biomass to that obtained from the Std medium (Holman-Sidak test: *t* = 1.415, *p* = 0.437). Similarly, the produced Chlo-a concentrations obtained from cells grown in the M7, M8, and M9 media were comparable to the concentration obtained from the Std medium (Holman-Sidak: *t* = 0.388, 1.593, and 0.691, respectively; *p* = 0.703, 0.424, and 0.748, respectively). Flow cytometric analysis of these samples verified these results and is presented in Figure 4B with the Chlo-a intensity of the bacterium grown in the Std medium in green as the reference.

**Figure 5:**
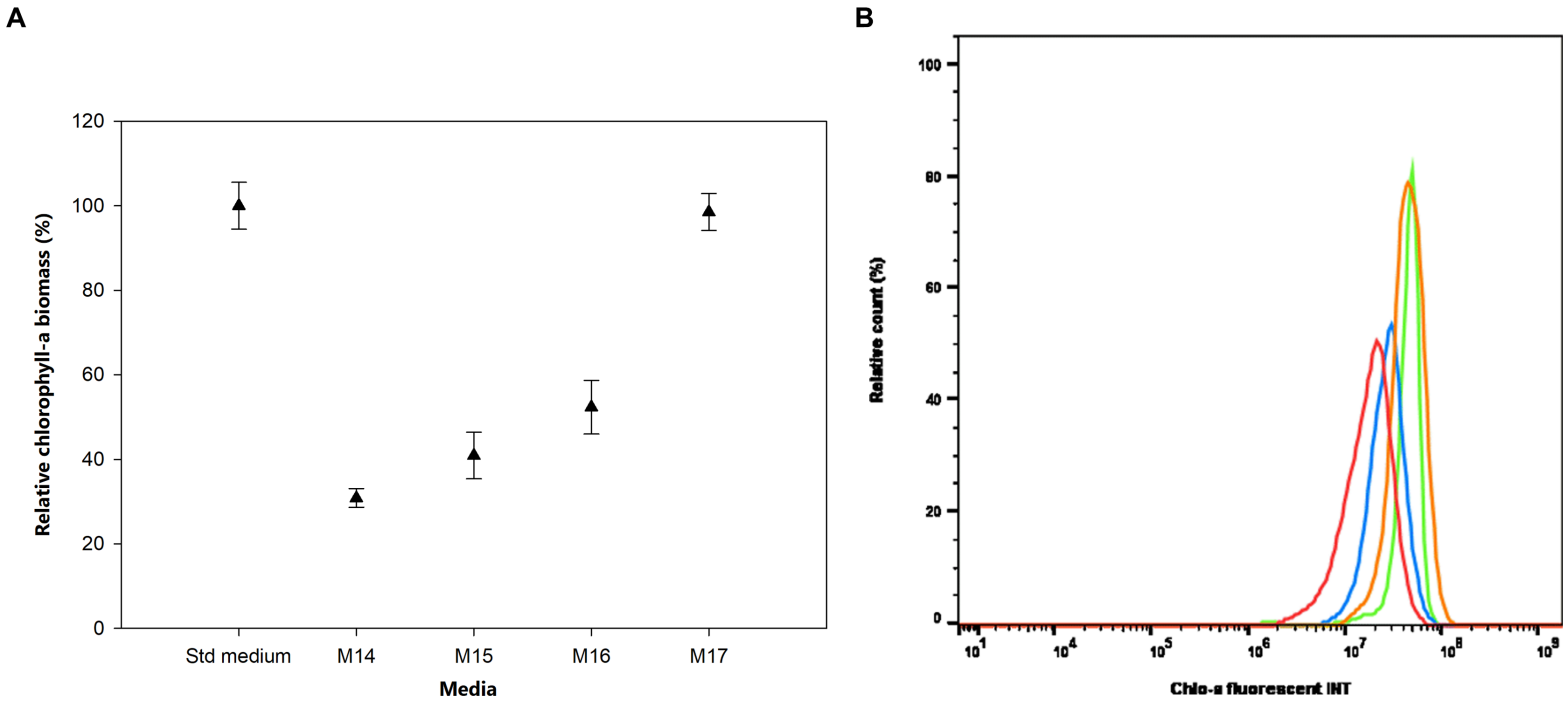
**(A)** Influence of sulfur deficiency on Chlo-a production of *Aphanizomenon* sp. ULC602, illustrated by means ± SD (*n* = 3). Statistical analysis was performed using one-way ANOVA (*n* = 15, *F* = 126.917, *p* < 0.001). **(B)** Flow cytometry analysis of *Aphanizomenon* sp. ULC602 in media containing various concentrations of SO_4_^2−^, Std medium (green); M14, containing 0 mM MgSO_4_ (no data); M15, containing 0.0015 mM MgSO_4_ (red); M16, containing 0.015 mM MgSO_4_ (blue); and M17, containing 0.15 mM MgSO_4_ (orange).

The effects of sulfur (S) deficiency were conducted by growing the bacterium in the M14 to M17 media with the concentration of S ranging from 0 to 0.15 mM. Figure 5 shows that at low concentrations of S in the M14 medium (0 mM), M15 medium (0.0015 mM), and M16 medium (0.015 mM) the bacterium produced less than 50% of the Chlo-a biomass compared to the Std medium containing 0.3 mM of MgSO_4_ (Holman-Sidak test: *t* = 16.891, 14.433, and 11.641, respectively; *p* < 0.05). The Chlo-a biomass obtained in the M17 medium, with 0.15 mM of the S source, was comparable with the amount produced in the Std medium (Holman-Sidak test: *t* = 0.361, *p* = 0.725). Furthermore, the bacterium could not produce Chlo-a in all media containing various forms and concentrations of phosphate (M20 to M23 media; illustrated in Supplementary Figure S1).

## 4 Discussion

In the present study, we evaluated the influences of growing parameters and essential nutrient sources on the growth of a bloom-forming filamentous cyanobacterium belonging to the *Aphanizomenon* genus, named *Aphanizomenon* sp. ULC602, which was recently isolated from a Belgian lake (Belgium, Europe) by BCCM. This strain was reported to contribute to the production of microcystin in Belgian waterbodies during summer (Van Hassel et al., 2022). The strain was cultivated and preserved at laboratory RT (23°C), which was reported as the normal temperature range in lakes and the favorable range for photosynthesis in most cyanobacteria, including *Anabaena, Microcystis,* and *Synechoccocus* (Konopka and Brock, 1978), as well as other studied strains of the *Aphanizomenon* genus (Cirés et al., 2013; Cirés and Ballot, 2016). The microscopic imaging indicated that the morphology of the studied bacterium was similar to that of other species in the *Aphanizomenon* genus with straight or bent shape, and trichomes were in a sub-symmetric structure consisting of cylindrical to barrel-shaped cells (Komárek and Komárková, 2006; Chorus and Welker, 2021). Spherical heterocysts were found intercalary between vegetative cells and sometimes at the terminal position (Komárek and Komárková, 2006). It was determined that the optimal photoperiod for the growth of cyanobacteria varies amongst each strain (Zevenboom and Mur, 1984; Jacob-Lopes et al., 2009; Polyzois et al., 2020). The target bacterium, *Aphanizomenon* sp. ULC602, was shown to grow faster and produce higher amounts of Chlo-a under a photoperiod of 24 h/0 h (light/dark) compared to the other tested periods (12 h/12 h and 0 h/24 h). The limitation of lighting time led to restraint of the utilization of inorganic carbon sources, thus reducing the energy for respiratory metabolism in cyanobacteria, which was assumed as the reason for this result (Jacob-Lopes et al., 2009). It can also explain the decrease in the growth and Chlo-a production in the media containing less inorganic carbon (M12 and M13) in this study.

Unlike previously studied species in the same genus and nitrogen-fixing species, which are grown in standard BG110 medium lacking a nitrogen source, *Aphanizomenon* sp. ULC602 was cultivated in a medium containing 50% dilution of the standard BG110 medium, indicating lower nutrient requirements for this strain compared to others. It was reported that the addition of N sources to the medium was demonstrated to improve the biomass of cyanobacteria (Cirés et al., 2017; Gour et al., 2018; Krausfeldt et al., 2019; Lu et al., 2019; Norena-Caro et al., 2021). The nitrogen can be supplied from atmospheric sources, from nitrates known as the popular form of dissolved inorganic nitrogen in aquatic systems, or from urea that is usually found in agro-industrial wastewaters (Glass et al., 2009); however, the presence of nitrogen sources from sodium nitrate (M10 medium) and urea (M11 medium) resulted in a decrease of the Chlo-a biomass produced by *Aphanizomenon* sp. ULC602 compared to the amount obtained in the Std medium, which was not observed for other cyanobacteria and *Aphanizomenon* strains in other studies. For instance, the growth of *Aphanizomenon gracile* was not influenced by various nitrate concentrations, while *Aphanizomenon aphanizomenoides* and *Aphanizomenon issatchenkoi* grew slowly in the condition of nitrate depletion (De Figueiredo et al., 2011). An increase in biomass of *Anabaena* sp. PCC7120 was seen in the presence of sodium nitrate and ammonium chloride in the media (Johnson et al., 2017). Similarly, the growth rate of *Anabaena* sp. UTEX 2576 in the presence of sodium nitrate was shown to be higher than in media with urea or without a nitrogen source, and the Chlo-a accumulation of this bacterium in sodium nitrate medium was also 50% higher than the latter media (Norena-Caro et al., 2021). By contrast, the growing inhibition of urea was observed with *Anabaena spiroides*, which is comparable with our target bacterium (Volk and Phinney, 1968). It is noted that the iron form used in the standard medium of *Aphanizomenon* sp. ULC602 is ammonium iron (III) citrate, which provides a small amount of nitrogen for the bacterium, creating a high C:N ratio in the formula of the medium. Supplementation of nitrogen in other forms led to a significant reduction of the C:N ratio in modified media. This could be the reason for the inhibition of the Chlo-a production of *Aphanizomenon* sp. ULC602.

Iron is an essential element for bacterial growth due to its role in the synthesis of DNA and sulfur proteins, such as nitrogenase, an important enzyme in nitrogen fixation (Walve and Larsson, 2007; Norena-Caro et al., 2021; Qiu et al., 2022). It was reported to be a limiting factor employed by photosynthetic organisms for their growth and metabolism (Keren et al., 2004; Qiu et al., 2022). Iron usually remains in aquatic systems in various forms including soluble inorganic and organic complexes (Qiu et al., 2022). It is demonstrated that iron stimulates not only nitrogenase activity but also the growth rate in cyanobacteria (Kumawat et al., 2006; Larson et al., 2018; Aslam et al., 2021; Norena-Caro et al., 2021). The supplementation of iron to the medium resulted in a remarkable rise in the numbers of trichomes and Chlo-a biomass since iron indirectly impacts chlorophyll synthesis by its precursor δ-aminolevulinic acid (ALA) (Kumawat et al., 2006; Wang et al., 2010). This could be the reason for the increase in the Chlo-a biomass produced by *Aphanizomenon* sp. ULC602 with the increase of the ferric (Fe^3+^) concentration in the medium (from 0 mM to 0.227 mM) under both studied forms (ammonium ferric citrate and ferric chloride); however, the amount of Chlo-a was saturated when the iron concentration was above 0.115 mM under different forms, including ferric or ferrous (FeSO_4_) forms. A similar observation of the Chlo-a saturation by iron concentration was noted in previous studies (Volk and Phinney, 1968; Wang et al., 2010). The Chlo-a biomass obtained from *Microcystis aeruginosa* reached the maximum level at the concentration of ammonium ferric citrate of 12.3 μmol Fe.L^−1^ (Wang et al., 2010). Notably, in the absence of a chelating agent (EDTA), or both EDTA and iron, the target bacterium cannot grow well. EDTA is a well-known chelating agent that plays a role in retaining the stability and bioavailability of iron in the medium due to its high affinity with iron, thus avoiding the precipitation of iron when it comes in contact with other elements (Martell et al., 1996; Oviedo and Rodríguez, 2003). Therefore, it can become evident that low Chlo-a synthesis of the target bacterium can be achieved when removing the chelating agent from its growing medium. The results also suggested that S deficiency caused the decrease in the bacterial growth and concentration of Chlo-a generated by *Aphanizomen* sp. ULC602, which was mentioned in other investigations with *Anabaena* species (Volk and Phinney, 1968; Kharwar and Mishra, 2020). The same trend was observed with the limitation of iron concentration since there was no significant effect on the bacterial growth and Chlo-a biomass with a concentration of MgSO_4_ higher than 0.15 mM. This result was similar to the S requirement of *Anabaeba spiroides* (Volk and Phinney, 1968). A study for the impact of long-term sulfur deficiency in *Anabaena* sp. PCC7120 suggested that S limitation causing the disruption of photosynthesis could be the reason for the reduction in biomass (Kharwar and Mishra, 2020). Interestingly, *Aphanizomenon* sp. ULC602 can grow slowly in the medium without phosphorous (P) source (M18 medium) but cannot grow well when increasing the concentration of P using potassium dihydrogen phosphate in the medium or replacing it with a low concentration of sodium dihydrogen phosphate (M19 to M21 media). Even though P is one of the major nutrient sources for the growth of cyanobacteria, the requirements for this source can be dependent on each species (Chen et al., 2020; Vuorio et al., 2020). Vuorio et al. (2020) evaluated phosphorus thresholds for several blooming cyanobacteria, in which the *Aphanizomenon* genus possesses the lowest threshold of 19 μg/L of total P.

## 5 Conclusion

Overall, the present work presents the growth and Chlo-a production of the freshwater cyanobacterium *Aphanizomenon* sp. ULC602 in different growing and nutrient conditions in modified media. Since the *Aphanizomenon* genus is one of the well-known producers of toxic bloom in water, this strain could be a candidate for the expansion of cyanotoxins in European freshwater lakes. Therefore, our investigation could contribute useful information to further studies for understanding its differential responses of growth to abiotic factors, thus controlling the explosion of this bacterium. This information could also be utilized to develop a nutrient-based biosensor for the detection of *Aphanizomenon* species in freshwater.

## Data availability statement

The original contributions presented in the study are included in the article/supplementary material, further inquiries can be directed to the corresponding author.

## Author contributions

M-LP and MB conceived the study and reviewed and edited the manuscript. M-LP designed the experiments and wrote the original draft of the manuscript. M-LP and EA performed the experiments and analyzed the data. MB contributed to project administration and funding acquisition. All authors have read and agreed to the published version of the manuscript.

## References

[ref1] Aslam A. Rasul S. Bahadar A. Hossain N. Saleem M. Hussain S.. (2021). Effect of micronutrient and hormone on microalgae growth assessment for biofuel feedstock. Sustainability 13:5035. doi: 10.3390/su13095035

[ref2] Ballot A. Fastner J. Lentz M. Wiedner C. (2010). First report of anatoxin-a-producing cyanobacterium Aphanizomenon issatschenkoi in northeastern Germany. Toxicon 56, 964–971. doi: 10.1016/j.toxicon.2010.06.021, PMID: 20615427

[ref3] Borowitzka Michael A. Moheimani Navid R. (2013): Algae for biofuels and energy. Dordrecht: Springer Developments in applied phycology, 5).

[ref4] Chen X. Dolinova I. Sevcu A. Jurczak T. Frankiewicz P. Wojtal-Frankiewicz A.. (2020). Strategies adopted by *Aphanizomenon flos-aquae* in response to phosphorus deficiency and their role on growth. Environ. Sci. Eur. 32:45. doi: 10.1186/s12302-020-00328-3

[ref5] Chorus Ingrid Welker Martin (2021): Toxic cyanobacteria in water. A guide to their public health consequences, monitoring and management. 2rd. Boca Raton: CRC Press.

[ref6] Cirés S. Ballot A. (2016). A review of the phylogeny, ecology and toxin production of bloom forming *Aphanizomenon* spp. and related species within the Nostocales (cyanobacteria). Harmful algae 54, 21–43. doi: 10.1016/j.hal.2015.09.007, PMID: 28073477

[ref7] Cirés S. Delgado A. González-Pleiter M. Quesada A. (2017). Temperature influences the production and transport of saxitoxin and the expression of sxt genes in the cyanobacterium *Aphanizomenon gracile*. Toxins 9:322. doi: 10.3390/toxins9100322, PMID: 29027918 PMC5666369

[ref8] Cirés S. Wörmer L. Wiedner C. Quesada A. (2013). Temperature-dependent dispersal strategies of *Aphanizomenon ovalisporum* (Nostocales, Cyanobacteria): implications for the annual life cycle. Microb. Ecol. 65, 12–21. doi: 10.1007/s00248-012-0109-8, PMID: 22915156

[ref9] Figueiredo D. R. De Goncalves A. M. M. Castro B. B. Goncalves F. Pereira M. J. Correia A. (2011). Differential inter- and intra-specific responses of *Aphanizomenon* strains to nutrient limitation and algal growth inhibition. J. Plankt. Res. 33, 1606–1616. doi: 10.1093/plankt/fbr058

[ref10] Glass J. B. Wolfe-Simon F. Anbar A. D. (2009). Coevolution of metal availability and nitrogen assimilation in cyanobacteria and algae. Geobiology 7, 100–123. doi: 10.1111/j.1472-4669.2009.00190.x, PMID: 19320747

[ref11] Gour R. S. Bairagi M. Garlapati V. K. Kant A. (2018). Enhanced microalgal lipid production with media engineering of potassium nitrate as a nitrogen source. Bioengineered 9, 98–107. doi: 10.1080/21655979.2017.1316440, PMID: 28471319 PMC5972933

[ref12] Grigoryeva N. Chistyakova L. (2020). “Confocal laser scanning microscopy for spectroscopic studies of living photosynthetic cells” in Color detection. eds. Zeng L.-W. Cao S.-L. (China: IntechOpen)

[ref13] Hallegraeff G. M. (1993). A review of harmful algal blooms and their apparent global increase. Phycologia 32, 79–99. doi: 10.2216/i0031-8884-32-2-79.1

[ref14] Hernández Mariné M. Clavero E. Roldan M. (2004). Microscopy methods applied to research on cyanobacteria. Limnetica 23, 179–186. doi: 10.23818/limn.23.16, PMID: 37505736

[ref15] Huang H. Wang W. Lv J. Liu Q. Liu X. Xie S.. (2022). Relationship between chlorophyll-a and environmental factors in lakes based on the random forest algorithm. Water 14:3128. doi: 10.3390/w14193128, PMID: 37906461

[ref16] Huisman J. Codd G. A. Paerl H. W. Ibelings B. W. Verspagen J. M. H. Visser P. M. (2018). Cyanobacterial blooms. Nat. Rev. Microb. 16, 471–483. doi: 10.1038/s41579-018-0040-1 29946124

[ref17] Jacob-Lopes E. Scoparo C. H. G. Lacerda L. M. C. F. Franco T. T. (2009). Effect of light cycles (night/day) on CO2 fixation and biomass production by microalgae in photobioreactors. J. CEP 48, 306–310. doi: 10.1016/j.cep.2008.04.007

[ref18] Jeong Y. Cho S.-H. Lee H. Choi H.-K. Kim D.-M. Lee C.-G.. (2020). Current status and future strategies to increase secondary metabolite production from Cyanobacteria. Microorganisms 8:1849. doi: 10.3390/microorganisms8121849, PMID: 33255283 PMC7761380

[ref19] Johnson T. J. Jahandideh A. Isaac I. C. Baldwin E. L. Muthukumarappan K. Zhou R.. (2017). Determining the optimal nitrogen source for large-scale cultivation of filamentous cyanobacteria. J. Appl. Phycol. 29, 1–13. doi: 10.1007/s10811-016-0923-3

[ref20] Kaplan-Levy Ruth N.; Hadas Ora Summers Michael L.; Rücker Jacqueline Sukenik Assaf (2010): Akinetes: dormant cells of cyanobacteria. In Lubzens Esther Cerda Joan Clark Melody (Eds.): Dormancy and resistance in harsh environments, Berlin, Heidelberg: Springer Berlin Heidelberg (Topics in Current Genetics).

[ref21] Keren N. Aurora R. Pakrasi H. B. (2004). Critical roles of bacterioferritins in iron storage and proliferation of Cyanobacteria. Plant physiol. 135, 1666–1673. doi: 10.1104/pp.104.042770, PMID: 15247377 PMC519080

[ref22] Kharwar S. Mishra A. K. (2020). Unraveling the complexities underlying sulfur deficiency and starvation in the cyanobacterium *Anabaena sp.* PCC 7120. Environ. Exp. Bot. 172:103966. doi: 10.1016/j.envexpbot.2019.103966

[ref23] Komárek J. Komárková J. (2006). Diversity of *Aphanizomenon*-like Cyanobacteria. Czech Phycology 6, 1–32.

[ref24] Konopka A. Brock T. D. (1978). Effect of temperature on blue-green algae(Cyanobacteria) in Lake Mendota. Appl. Envir. Microbiol. 36, 572–576. doi: 10.1128/aem.36.4.572-576.1978, PMID: 16345318 PMC243093

[ref25] Krausfeldt L. E. Farmer A. T. Gonzalez C. Hector F. Zepernick B. N. Campagna S. R.. (2019). Urea is both a carbon and nitrogen source for *Microcystis aeruginosa:* tracking 13C incorporation at bloom pH conditions. Front. Microbiol. 10:1064. doi: 10.3389/fmicb.2019.01064 31164875 PMC6536089

[ref26] Kultschar B. Llewellyn C. (2018). “Secondary metabolites in Cyanobacteria” in Secondary metabolites - sources and applications: InTech. eds. Vijayakumar R. Raja S. S. S. (Crotia: InTech – Open Science)

[ref27] Kumar K. Mella-Herrera R. A. Golden J. W. (2009). Cyanobacterial heterocysts. Cold Spring Harb. Perspect. Biol. 2:a000315. doi: 10.1101/cshperspect.a000315 PMC2845205 20452939

[ref28] Kumawat R. N. Rathore P. S. Nathawat N. S. Mahatma M. (2006). Effect of sulfur and iron on enzymatic activity and chlorophyll content of mungbean. J. Plant. Nutr. 29, 1451–1467. doi: 10.1080/01904160600837162

[ref29] Larson C. A. Mirza B. Rodrigues J. L. M. Passy S. I. (2018). Iron limitation effects on nitrogen-fixing organisms with possible implications for cyanobacterial blooms. FEMS Microbiol. Eco. 94, 1–8. doi: 10.1093/femsec/fiy046 29566225

[ref30] Lu J. Zhu B. Struewing I. Xu N. Duan S. (2019). Nitrogen–phosphorus-associated metabolic activities during the development of a cyanobacterial bloom revealed by metatranscriptomics. Sci. Reports 9:2480. doi: 10.1038/s41598-019-38481-2 PMC6385219 30792397

[ref31] Martell A. E. Motekaitis R. J. Chen D. Hancock R. D. McManus D. (1996). Selection of new Fe(III)/Fe(II) chelating agents as catalysts for the oxidation of hydrogen sulfide to sulfur by air. Can. J. Chem. 74, 1872–1879. doi: 10.1139/v96-210

[ref32] Mehdizadeh Allaf M. Peerhossaini H. (2022). Cyanobacteria: model microorganisms and beyond. Microorganisms 10:696. doi: 10.3390/microorganisms10040696, PMID: 35456747 PMC9025173

[ref33] Moore S. K. Trainer V. L. Mantua N. J. Parker M. S. Laws E. A. Backer L. C.. (2008). Impacts of climate variability and future climate change on harmful algal blooms and human health. Environ. Health 7:S4. doi: 10.1186/1476-069X-7-S2-S4, PMID: 19025675 PMC2586717

[ref34] Mudaliar A. Pandya U. (2023). Assessment of cyanobacterial chlorophyll a as an Indicator of water quality in two wetlands using multi-temporal sentinel-2 images. In Environ. Sci. Proc. 25:68. doi: 10.3390/ECWS-7-14252

[ref35] Nairn R. C. (Ed.) (1969): Fluorescent protein tracing. 3rd. Baltimore: Williams and Wilkins.

[ref36] Norena-Caro D. A. Malone T. M. Benton M. G. (2021). Nitrogen sources and iron availability affect pigment biosynthesis and nutrient consumption in *Anabaena sp*. UTEX 2576. Microorganisms 9:431. doi: 10.3390/microorganisms9020431, PMID: 33669780 PMC7922959

[ref37] Oviedo C. Rodríguez J. (2003). EDTA: the chelating agent under environmental scrutiny. Quím. Nova 26, 901–905. doi: 10.1590/S0100-40422003000600020, PMID: 12380068

[ref38] Pham M.-L. Tran A.-M. Mathiesen G. Nguyen H.-M. Nguyen T.-H. (2020). Cell wall anchoring of a bacterial chitosanase in *Lactobacillus plantarum u*sing a food-grade expression system and two versions of an LP × TG anchor. IJMS 21:3773. doi: 10.3390/ijms21113773 32471049 PMC7312796

[ref39] Polyzois A. Kirilovsky D. Dufat T.-H. Michel S. (2020). Effects of modification of light parameters on the production of cryptophycin, cyanotoxin with potent anticancer activity, in *Nostoc* sp. Toxins 12:809. doi: 10.3390/toxins12120809, PMID: 33371249 PMC7766261

[ref40] Preußel K. Wessel G. Fastner J. Chorus I. (2009). Response of cylindrospermopsin production and release in *Aphanizomenon flos-aquae* (Cyanobacteria) to varying light and temperature conditions. Hamful Algae 9, 645–650. doi: 10.1016/j.hal.2008.10.009

[ref41] Qiu G.-W. Koedooder C. Qiu B.-S. Shaked Y. Keren N. (2022). Iron transport in Cyanobacteria - from molecules to communities. Trends Microb. 30, 229–240. doi: 10.1016/j.tim.2021.06.001, PMID: 34175176

[ref42] Rastogi R. P. Madamwar D. Incharoensakdi A. (2015). Bloom dynamics of Cyanobacteria and their toxins: environmental health impacts and mitigation strategies. Front. Microbiol. 6:1254. doi: 10.3389/fmicb.2015.01254, PMID: 26635737 PMC4646972

[ref43] Rekar S. Hindák F. (2002). *Aphanizomenon slovenicum* sp. nov.: morphological and ecological characters of a new cyanophyte/cyanobacterial species from Lake bled, Slovenia. Ann. Limnol. 38, 271–285. doi: 10.1051/limn/2002022

[ref44] Singh Pratika Khan Azmi Srivastava Amrita (2020): Heterocyst and akinete differentiation in cyanobacteria: a view toward cyanobacterial symbiosis. In: Advances in Cyanobacterial biology. India: Elsevier, Academic Press. pp. 235–248.

[ref45] Singh S. P. Montgomery B. L. (2011). Determining cell shape: adaptive regulation of cyanobacterial cellular differentiation and morphology. Trends Microbiol. 19, 278–285. doi: 10.1016/j.tim.2011.03.001, PMID: 21458273

[ref46] Üveges V. Tapolczai K. Krienitz L. Padisák J. (2012). Photosynthetic characteristics and physiological plasticity of an *Aphanizomenon flos-aquae* (Cyanobacteria, Nostocaceae) winter bloom in a deep oligo-mesotrophic Lake (Lake Stechlin, Germany). Hydrobiologia 698, 263–272. doi: 10.1007/s10750-012-1103-3

[ref47] Van Hassel W. H. R. Andjelkovic M. Durieu B. Marroquin V. A. Masquelier J. Huybrechts B.. (2022). A summer of cyanobacterial blooms in Belgian waterbodies: microcystin quantification and molecular characterizations. Toxins 14:61. doi: 10.3390/toxins14010061, PMID: 35051038 PMC8780180

[ref48] Viso-Vázquez M. Acuña-Alonso C. Rodríguez J. L. Álvarez X. (2021). Remote detection of cyanobacterial blooms and chlorophyll-a analysis in a eutrophic reservoir using sentinel-2. Sustainability 13:8570. doi: 10.3390/su13158570

[ref49] Volk S. L. Phinney H. K. (1968). Mineral requirements for the growth of *Anabaena spiroides* in vitro. Canadian J. Botany 44:619. doi: 10.1139/b68-090

[ref50] Vuorio K. Järvinen M. Kotamäki N. (2020). Phosphorus thresholds for bloom-forming cyanobacterial taxa in boreal lakes. Hydrobiologia 847, 4389–4400. doi: 10.1007/s10750-019-04161-5

[ref51] Walve J. Larsson U. (2007). Blooms of Baltic Sea *Aphanizomenon* sp. (Cyanobacteria) collapse after internal phosphorus depletion. Aquat. Microb. Ecol. 49, 57–69. doi: 10.3354/ame01130

[ref52] Wang C. Kong H.-N. Wang X.-Z. Wu H.-D. Lin Y. HE S.-B. (2010). Effects of iron on growth and intracellular chemical contents of *Microcystis aeruginosa*. Biomed. Environ. Sci. 23, 48–52. doi: 10.1016/S0895-3988(10)60031-1, PMID: 20486436

[ref53] Wen Q. Xiao P. Li H. Li W. Yu G. Li R. (2022). Succession of *Aphanizomenon flos-aquae* and *Microcystis aeruginosa* in direct co-culture experiments at different temperatures and biomasses. J. Oceanol. Limnol. 40, 1819–1828. doi: 10.1007/s00343-022-2041-1

[ref54] Zavřel T. Sinetova M. A. Červený J. (2015). Measurement of chlorophyll-a and carotenoids concentration in Cyanobacteria. Bio-protocol 5, 1–5. doi: 10.21769/BioProtoc.1467

[ref55] Zevenboom W. Mur L. R. (1984). Growth and photosynthetic response of the cyanobacterium *Microcystis aeruginosa* in relation to photoperiodicity and irradiance. Archiv Microb 139, 232–239. doi: 10.1007/BF00402006

[ref56] Zhang X. Jia X. Yan L. Wang J. Kang X. Cui L. (2017). Cyanobacterial nitrogen fixation influences the nitrogen removal efficiency in a constructed wetland. Water 9:865. doi: 10.3390/w9110865

