# Supplementary material for: Growth of freshwater cyanobacterium Aphanizomenon sp. ULC602 in different growing and nutrient conditions
Source: Front Microbiol. 2023 Dec 21;14:1220818. doi: 10.3389/fmicb.2023.1220818 (PMC10768055; doi:10.3389/fmicb.2023.1220818)
Supplement: Supplementary file 1 [file Data_Sheet_1.docx]

Supplementary Material

# Supplementary Tables

**Table S1: Components of modified 50%BG110 media in 1L**

| **Component (mM)** |  | **NaNO_3_** | **K_2_HPO_4._3H_2_O** | **MgSO_4_.7H_2_O** | **CaCl_2_.2H_2_O** | **EDTA -Mg- Na_2_** | **Citric acid (monohydrate)** | **Ammonium iron citrate** | **Na_2_CO_3_** | **NaHCO_3_** | **Trace elements (in ml)** | **FeCl3.6H2O** | **FeSO4. 7H2O** | **urea** | **KH2PO4** | **NaH2PO4** |
| --- | --- | --- | --- | --- | --- | --- | --- | --- | --- | --- | --- | --- | --- | --- | --- | --- |
| **Modified media** | **Std** | 0.00 | 0.088 | 0.31 | 0.12 | 0.0013 | 0.015 | 0.0115 | 0.095 | 0.900 | 0.500 | 0 | 0 | 0 | 0 | 0 |
|  | **M2** | 0.00 | 0.088 | 0.31 | 0.12 | 0.0013 | 0.015 | 0 | 0.095 | 0.900 | 0.500 | 0 | 0 | 0 | 0 | 0 |
|  | **M3** | 0.00 | 0.088 | 0.31 | 0.12 | 0 | 0.015 | 0 | 0.095 | 0.900 | 0.500 | 0 | 0 | 0 | 0 | 0 |
|  | **M4** | 0.00 | 0.088 | 0.31 | 0.12 | 0.0013 | 0.015 | 0 | 0.095 | 0.900 | 0.500 | 0.000185 | 0 | 0 | 0 | 0 |
|  | **M5** | 0.00 | 0.088 | 0.31 | 0.12 | 0.0013 | 0.015 | 0.0115 | 0.095 | 0.900 | 0.500 | 0.000185 | 0 | 0 | 0 | 0 |
|  | **M6** | 0.00 | 0.088 | 0.31 | 0.12 | 0.0013 | 0.015 | 0 | 0.095 | 0.900 | 0.500 | 0 | 0.0359 | 0 | 0 | 0 |
|  | **M7** | 0.00 | 0.088 | 0.31 | 0.12 | 0.0013 | 0.015 | 0.00566 | 0.095 | 0.900 | 0.500 | 0 | 0 | 0 | 0 | 0 |
|  | **M8** | 0.00 | 0.088 | 0.31 | 0.12 | 0.0013 | 0.015 | 0.02265 | 0.095 | 0.900 | 0.500 | 0 | 0 | 0 | 0 | 0 |
|  | **M9** | 0.00 | 0.088 | 0.31 | 0.12 | 0.0013 | 0.015 | 0.0115 | 0.095 | 0.900 | 0.500 | 0 | 0.0359 | 0 | 0 | 0 |
|  | **M10** | 8.83 | 0.088 | 0.31 | 0.12 | 0.0013 | 0.015 | 0.0115 | 0.095 | 0.900 | 0.500 | 0 | 0 | 0 | 0 | 0 |
|  | **M11** | 0 | 0.088 | 0.31 | 0.12 | 0.0013 | 0.015 | 0.0115 | 0.095 | 0.900 | 0.500 | 0 | 0 | 1.5 | 0 | 0 |
|  | **M12** | 0 | 0.088 | 0.31 | 0.12 | 0.0013 | 0.015 | 0.0115 | 0 | 0 | 0.500 | 0 | 0 | 0 | 0 | 0 |
|  | **M13** | 0 | 0.088 | 0.31 | 0.12 | 0.0013 | 0.015 | 0.0115 | 0.095 | 0 | 0.500 | 0 | 0 | 0 | 0 | 0 |
|  | **M14** | 0 | 0.088 | 0 | 0.12 | 0.0013 | 0.015 | 0.0115 | 0.095 | 0.9 | 0.500 | 0 | 0 | 0 | 0 | 0 |
|  | **M15** | 0 | 0.088 | 0.0015 | 0.12 | 0.0013 | 0.015 | 0.0115 | 0.095 | 0.9 | 0.500 | 0 | 0 | 0 | 0 | 0 |
|  | **M16** | 0 | 0.088 | 0.015 | 0.12 | 0.0013 | 0.015 | 0.0115 | 0.095 | 0.9 | 0.500 | 0 | 0 | 0 | 0 | 0 |
|  | **M17** | 0 | 0.088 | 0.15 | 0.12 | 0.0013 | 0.015 | 0.0115 | 0.095 | 0.9 | 0.500 | 0 | 0 | 0 | 0 | 0 |
|  | **M18** | 0 | 0 | 0.31 | 0.12 | 0.0013 | 0.015 | 0.0115 | 0.095 | 0.9 | 0.500 | 0 | 0 | 0 | 0 | 0 |
|  | **M19** | 0 | 0.175 | 0.31 | 0.12 | 0.0013 | 0.015 | 0.0115 | 0.095 | 0.9 | 0.500 | 0 | 0 | 0 | 0.65 | 0 |
|  | **M20** | 0 | 0 | 0.31 | 0.12 | 0.0013 | 0.015 | 0.0115 | 0.095 | 0.9 | 0.500 | 0 | 0 | 0 | 0 | 0.018 |
|  | **M21** | 0 | 1.45 | 0.31 | 0.12 | 0.0013 | 0.015 | 0.0115 | 0.095 | 0.9 | 0.500 | 0 | 0 | 0 | 0 | 0 |

# Supplementary Figures


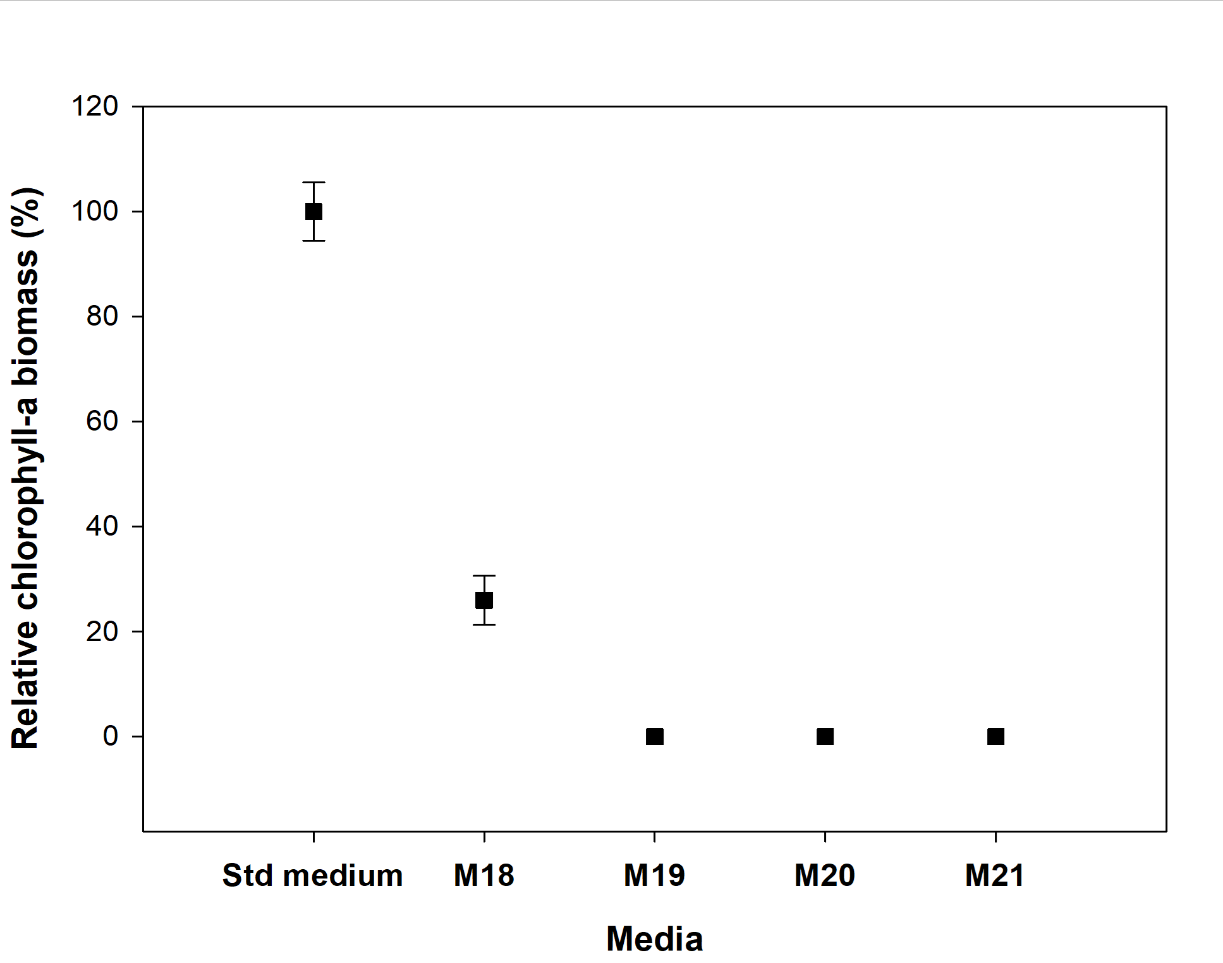


**Figure S1**: Effects of phosphorus (P) sources to Chlo-a production of *Aphanizomenon* sp*.* ULC602 in Std medium and modified 50% BG110 media: M18: containing no P-sources; M19: containing K_2_PO_4_ 0.175 mM and KH_2_PO_4_ 0.65 mM; M20: containing NaH_2_PO_4_ 0.018 mM; M21: containing K_2_HPO_4_ 1.45 mM. Data was presented as the mean ± SD (n=3). Statistical analysis was performed by one-way ANOVA with n=15; *F*=533.671, *P*<0.001. The significant differences between each treatment and the Std medium were tested by Holman-Sidak test with *P*<0.05 in all cases.
